# Supplementary material for: Temperature-Dependent Development of Nitidula rufipes (Linnaeus, 1767) (Coleoptera: Nitidulidae) and Its Significance in Estimating Minimum Postmortem Interval
Source: Insects. 2023 Mar 20;14(3):299. doi: 10.3390/insects14030299 (PMC10058101; doi:10.3390/insects14030299)
Supplement: Supplementary file 1 [file insects-14-00299-s001.zip › Table S5.docx]

| **Table S5.** Nitidulidae with species identified and their ecological niche information in nearly 30 years of succession studies and case reports in forensic entomology. | | | | | | | |
| --- | --- | --- | --- | --- | --- | --- | --- |
| Genera | Species | Data sources | DAT_min_/DAT_max_ | Months | Habitats | Carrion | Decomposition  stages |
| *Omosita* | *Omosita colon* | Lyu et al. [1] | 13.0°C/36.0°C | March to October | Forest | Pig | F to R |
|  |  | Hu et al. [2] | 15.0°C/32.9°C | July to September | Rural area | Pig | AD to R |
|  |  | Anton et al. [3] | -/- | April to August | Limestone hillsides | Pig | B |
|  |  | Kočárek [4] | -/- | May to August | Meadow and forest | Rat | D to R |
|  |  | Matuszewski et al. [5] | 10.5°C/28.3°C | May to August | Grassland | Pig | - |
|  |  | De Jong and Hoback [6] | 20.7°C/39.3°C | June to August | Field | Rat | - |
|  |  | Matuszewski et al. [7] | 7.0°C/19.0°C | April to June | Forest | Pig | - |
|  |  | Watson and Carlton [8] | --/- | October to March | Forest | Bear、deer、alligator and pig | - |
|  |  | Matuszewski et al. [9] | -/- | April to July | Grassland and forest | Pig | D to R |
|  |  | Diaz-Aranda et al. [10] | -/- | - | Natural habitats | Pig and sheep | - |
|  |  | Bourel et al. [11] | -/- | May to July | Grassland and wood | Rabbit | - |
|  |  | Williams et al. [12] | -/- | May to August | Natural Preserves | Chicken and fish | - |
|  |  | Saloña et al. [13] | 12.0°C/31.0°C | August | Forest | Human | AD |
|  |  | Watson and Carlton [14] | -/- | April to July | Forest | Bear、deer、alligator and pig | - |
|  |  | Li et al. [15] | 12.6°C/30.2°C | April to September | Shrubs | Pig | AD |
|  |  | Williams et al.[16] | -/- | February to March | Rubbish dumps | Sheep | - |
|  |  | Pastula and Merritt [17] | -/- | July | Grassland | Pig | - |
|  |  | Park et al. [18] | 14.8°C/42.0°C | April to November | Hillside | Pig | F to R |
|  |  | Probst et al. [19] | -/- | - | Forest | Pig | - |
|  |  | Lutz et al. [20] | -/- | June to September | Field | Pig | - |
|  |  | Perez et al. [21] | -/- | June to August | Woodlots | Pig | - |
|  |  | Michaud and Moreau [22] | -/- | May to November | Forest | Pig | - |
|  | *Omosita discoidea* | Lyu et al. [1] | 26.0°C/36.0°C | June to August | Forest | Pig | F to R |
|  |  | Anton et al. [3] | -/- | April to May | Limestone hillsides | Pig | B |
|  |  | Kočárek [4] | -/- | May to November | Forest | Rat | D to R |
|  |  | Matuszewski et al. [7] | 7.0°C/23.0°C | April to August | Forest | Pig | - |
|  |  | Matuszewski et al. [9] | -/- | June | Forest | Pig | - |
|  |  | Bourel et al. [11] | -/- | May to July | Wood | Rabbit |  |
|  |  | Park et al. [18] | 14.8°C/38.9°C | April to June | Hillside | Pig | B to R |
|  |  | Jarmusz et al. [23] | -/- | April to June | Forest | Pig |  |
|  |  | Michaud and Moreau [22] | -/- | May to November | Forest | Pig | - |
|  | *Omosita depressa* | Kočárek [4] | -/- | May to November | Forest | Rat | D to R |
|  |  | Matuszewski et al. [7] | 7.0°C/19.0°C | April to June | Forest | Pig | - |
|  |  | Matuszewski et al. [9] | -/- | June to August | Grassland and forest | Pig | - |
|  |  | Saloña et al. [13] | 12.0°C/31.0°C | August | Forest | Human | AD |
|  |  | Jarmusz et al. [23] | -/- | April to August | Forest | Pig |  |
|  | *Omosita nearctica* | Williams et al. [16] | -/- | February to March | Rubbish dumps | Sheep | - |
|  | *Omosita japonica* | Park et al. [18] | 14.8°C/42.0°C | April to November | Hillside | Pig | F to R |
| *Nitidula* | *Nitidula carnaria* | Hu et al. [2] | 15.0°C/32.9°C | July to September | Rural area | Pig | AD to R |
|  |  | Matuszewski et al. [5] | 10.5°C/28.3°C | May to August | Grassland | Pig | - |
|  |  | Matuszewski et al. [9] | -/- | April to July | Grassland | Pig | D to R |
|  |  | Baz et al. [24] | 8.6°C/21.5°C | March to September | Periurban | Squid | - |
|  |  | Diaz-Aranda et al. [25] | -4.0°C/35.0°C | Four seasons | Uncultivated plot | Pig | AC to R |
|  |  | Zanetti et al. [26] | 3.8°C/29.4°C | Four seasons | Field | Pig | AC to R |
|  |  | Ozdemir and Sert [27] | 17.0°C/35.0°C | April to October | Wood | Pig | B to R |
|  |  | Bajerlein et al. [28] | -/- | July | Suburb | Human | AD |
|  |  | Ortloff et al. [29] | 5.1°C/30.8°C | November | Slopes of mountain | Pig | AD |
|  |  | Martin-Vega et al. [30] | -/- | Four seasons | Rural area | Pig | - |
|  |  | Matuszewski and Mądra-Bielewicz [31] | 15.5°C/23.5°C | August | Suburbs | Human | AD to R |
|  | *Nitidula rufipes* | Hu et al. [2] | 15.0°C/32.9°C | July to September | Rural area | Pig | AD to R |
|  |  | Anton et al. [3] | -/- | June | Limestone hillsides | Pig | - |
|  |  | Matuszewski et al. [5] | 10.5°C/28.3°C | May to August | Grassland | Pig | - |
|  |  | Matuszewski et al. [9] | -/- | April | Grassland | Pig | D to R |
|  |  | Bourel et al. [11] | -/- | May to July | Grassland | Rabbit | - |
|  |  | Li et al. [15] | -/- | April to September | Shrubs | Pig | AC |
|  |  | Diaz-Aranda et al. [25] | -4.0°C/35.0°C | Four seasons | Uncultivated plot | Pig | AC to R |
|  |  | Ozdemir and Sert [27] | 15.0°C/32.0°C | April to July and October | Wood | Pig | AC to R |
|  |  | Park et al. [18] | 14.8°C/38.9°C | April to June | Hillside | Pig | B to R |
|  |  | Martin-Vega et al. [30] | -/- | January to May | Rural area | Pig | - |
|  |  | Michaud and Moreau [22] | -/- | May to November | Forest | Pig | - |
|  | *Nitidula bipunctata* | Anton et al. [3] | 12.6°C/30.2°C | April to June | Limestone hillsides | Pig | B to D |
|  |  | Kočárek [4] | -/- | May to August | Meadow and forest | Rat | D to R |
|  |  | Matuszewski et al. [9] | -/- | April to June | Grassland and forest | Pig | D to R |
|  |  | Mashaly et al. [32] | 7.0°C/21.4°C | January | Underground in sandy soils | Human | R |
|  |  | Pérez-Marcos et al. [33] | 18.0°C/26.0°C | September to October | Mountain | Pig | R |
|  |  | Michaud and Moreau [22] | -/- | May to November | Forest | Pig | - |
|  | *Nitidula flavomaculata* | Diaz-Aranda et al. [10] | -/- | - | Natural habitats | Pig and sheep | - |
|  |  | Diaz-Aranda et al. [25] | -4.0°C/35.0°C | Four seasons | Uncultivated plot | Pig | AC to R |
|  |  | Ozdemir and Sert [27] | 10.0°C/28.0°C | April to May  September to November | Wood | Pig | B to R |
|  |  | Moemenbellah-Fard et al. [34] | -/- | December | Mountain field | Human | R |
|  |  | Adair and Kondratieff [35] | -/- | January | Field | Human | D |
|  |  | Bonacci et al. [36] | 6.0°C/23.0°C | February to May | Wild meadow | Pig | B to R |
|  |  | Bonacci et al.[37] | 5.6°C/26.5°C | - | Underground | Pig | AC to R |
|  |  | Martin-Vega et al. [30] | -/- | Four seasons | Rural area | Pig | - |
|  | *Nitidula ziczac Say* | De Jong and Hoback [6] | 20.7°C/39.3°C | June to August | Field | Rat | - |
| *Carpophilus* | *Carpophilus marginellus* | Park et al. [18] | 26.0°C/42.0°C | June to August | Hillside | Pig | F to R |
|  | *Carpophilus hemipterus* | Oliva (2001)[38] | 11.4°C/30.2°C | - | Rural area | Beef | - |
| *Glischrochilus* | *Glischrochilus ipsodes* | Jung et al. (2008)[39] | 16.0°C/24.0°C | R | May | Mountain | Pig |

The topics "forensic entomology and succession" and "forensic entomology and case" were searched in the database Web of Science, and the checklist of species in relevant search results was viewed in turn, a total of 39 literature mentioned Nitidulidae with species identified and the corresponding information was extracted. T_min_ represents minimum daily average temperature, and T_max_ represents maximum daily average temperature. F indicates fresh stage, B indicates bloated stage, AC indicates active decay stage, AD indicates advanced stage, D indicates decay stage, and R indicates remains/dry/mummified stage.

**References**

1. Lyu, Z.; Wan, L.H.; Yang, Y.Q.; Tang, R.; Xu, L.Z. A checklist of beetles (Insecta, Coleoptera) on pig carcasses in the suburban area of southwestern China: A preliminary study and its forensic relevance. *J. Forensic Leg. Med.* **2016**, *41*, 42-48.

2. Hu, G.W.; Kang, C.T.; Zhu, R.; Guo, Y.; Li, L.L.; Wang, Y.H.; Zhang, Y.N.; Wang, Y.; Wang, J.F. A preliminary study of body decomposition and arthropod succession in an arid area in Northwest China during summer. *J. Med. Entomol.* **2023**, *60*, 306–315.

3. Anton, E.; Niederegger, S.; Beutel, R.G. Beetles and flies collected on pig carrion in an experimental setting in Thuringia and their forensic implications. *Med. Vet. Entomol.* **2011**, *25*, 353-364.

4. Kočárek, P. Decomposition and Coleoptera succession on exposed carrion of small mammal in Opava, the Czech Republic. *Eur. J. Soil Biol.* **2003**, *39*, 31-45.

5. Matuszewski, S.; Frątczak, K.; Konwerski, S.; Bajerlein, D.; Szpila, K.; Jarmusz, M.; Szafałowicz, M.; Grzywacz, A.; Mądra, A. Effect of body mass and clothing on carrion entomofauna. *Int. J. Legal Med.* **2016**, *130*, 221-232.

6. De Jong, G.D.; Hoback, W.W. Effect of investigator disturbance in experimental forensic entomology: succession and community composition. *Med. Vet. Entomol.* **2006**, *20*, 248-258.

7. Matuszewski, S.; Bajerlein, D.; Konwerski, S.; Szpila, K. Insect succession and carrion decomposition in selected forests of Central Europe. Part 2: Composition and residency patterns of carrion fauna. *Forensic Sci. Int.* **2010**, *195*, 42-51.

8. Watson, E.J.; Carlton, C.E. Insect succession and decomposition of wildlife carcasses during fall and winter in Louisiana. *J. Med. Entomol.* **2005**, *42*, 193-203.

9. Matuszewski, S.; Szafałowicz, M.; Jarmusz, M. Insects colonising carcasses in open and forest habitats of Central Europe: search for indicators of corpse relocation. *Forensic Sci. Int.* **2013**, *231*, 234-239.

10. Díaz-Aranda, L.M.; Martín-Vega, D.; Baz, A.; Cifrián, B. Larval identification key to necrophagous Coleoptera of medico-legal importance in the western Palaearctic. *Int. J. Legal Med.* **2018**, *132*, 1795-1804.

11. Bourel, B.; Martin-Bouyer, L.; Hedouin, V.; Cailliez, J.C.; Derout, D.; Gosset, D. Necrophilous insect succession on rabbit carrion in sand dune habitats in northern France. *J. Med. Entomol.* **1999**, *36*, 420-425.

12. Williams, R.N.; Blackmer, J.L.; Richmond, D.S.; Ellis, M.S. Nitidulidae (Coleoptera) diversity in three natural preserves in Portage County, Ohio. **1992**, *92*,82-87.

13. Saloña, M.I.; Moraza, M.L.; Carles Tolrá, M.; Iraola, V.; Bahillo, P.; Yélamos, T.; Outerelo, R.; Alcaraz, R. Searching the soil: forensic importance of edaphic fauna after the removal of a corpse. *J. Forensic Sci.* **2010**, *55*, 1652-1655.

14. Watson, E.J.; Carlton, C.E. Spring succession of necrophilous insects on wildlife carcasses in Louisiana. *J. Med. Entomol.* **2003**, *40*, 338-347.

15. Li, L.L.; Guo, Y.; Zhou, Y.X.; Yang, Y.; Kang, C.T.; Hu, G.W.; Wang, Y.H.; Zhang, Y.N.; Wang, Y.; Wang, J.F. Succession patterns of sarcosaprophagous insects on pig carcasses in different months in Yangtze River Delta, China. *Forensic Sci. Int.* **2022**, *342*, 111518.

16. Williams, K.A.; Clitheroe, C.L.; Villet, M.H.; Midgley, J.M. The first record of *Omosita nearctica* Kirejtshuk (Coleoptera, Nitidulidae) in South Africa, with the first description of its mature larva. *Afr. Invertebr.* **2021**, *62*, 257-271.

17. Pastula, E.C.; Merritt, R.W. Insect arrival pattern and succession on buried carrion in Michigan. *J. Med. Entomol.* **2013**, *50*, 432-439.

18. Park, S.H.; Lee, J.H.; Woo, D.; Ji, B.H.; Moon, T.Y. Insect diversity and succession patterns on pig cadavers in Changwon, South Korea. *Entomol. Res.* **2022**, *52*, 241-250.

19. Probst, C.; Gethmann, J.; Amendt, J.; Lutz, L.; Teifke, J.P.; Conraths, F.J. Estimating the postmortem interval of wild boar carcasses. *Vet. Sci.* **2020**, *7*, 6.

20. Lutz, L.; Amendt, J.; Moreau, G. Carcass concealment alters assemblages and reproduction of forensically important beetles. *Forensic Sci. Int.* **2018**, *291*, 124-132. 92.

21. Perez, A.E.; Haskell, N.H.; Wells, J.D. Evaluating the utility of hexapod species for calculating a confidence interval about a succession based postmortem interval estimate. *Forensic Sci. Int.* **2014**, *241*, 91–95.

22. Michaud, J.P.; Majka, C.G.; Privé, J.P.; Moreau, G. Natural and anthropogenic changes in the insect fauna associated with carcasses in the North American Maritime lowlands. *Forensic Sci. Int.* **2010**, *202*, 64-70.

23. Jarmusz, M.; Grzywacz, A.; Bajerlein, D. A comparative study of the entomofauna (Coleoptera, Diptera) associated with hanging and ground pig carcasses in a forest habitat of Poland - ScienceDirect. *Forensic Sci. Int.* **2020**, *309,* 110212.

24. Baz, A.; Botías, C.; Martín-Vega, D.; Cifrián, B.; Díaz-Aranda, L.M. Preliminary data on carrion insects in urban (indoor and outdoor) and periurban environments in central Spain. *Forensic Sci. Int.* **2015**, *248*, 41-47.

25. Díaz-Aranda, L.M.; Martín-Vega, D.; Gómez-Gómez, A.; Cifrián, B.; Baz, A. Annual variation in decomposition and insect succession at a periurban area of central Iberian Peninsula. *J. Forensic Leg. Med.* **2018**, *56*, 21-31.

26. Zanetti, N.I.; Visciarelli, E.C.; Centeno, N.D. Associational patterns of scavenger beetles to decomposition stages. *J. Forensic Sci.* **2015**, *60*, 919-927.

27. Özdemir, S.; Sert, O. Determination of Coleoptera fauna on carcasses in Ankara province, Turkey. *Forensic Sci. Int.* **2009**, *183*, 24-32.

28. Bajerlein, D.; Taberski, D.; Matuszewski, S. Estimation of postmortem interval (PMI) based on empty puparia of *Phormia regina* (Meigen) (Diptera: Calliphoridae) and third larval stage of *Necrodes littoralis* (L.) (Coleoptera: Silphidae)–Advantages of using different PMI indicators. *J. Forensic Leg. Med.* **2018**, *59*, 59.

29. Ortloff, A.; Zanetti, N.; Centeno, N.; Silva, R.; Bustamante, F.; Olave, A. Ultramorphological characteristics of mature larvae of *Nitidula carnaria* (Schaller 1783) (Coleoptera: Nitidulidae), a beetle species of forensic importance. *Forensic Sci. Int.* **2014**, *239*, e1-e9.

30. Martín-Vega, D.; Baz, A.; Cifrián, B.; Gómez-Gómez, A.; Díaz-Aranda, L.M. Long-term insect successional patterns on pig carcasses in central Spain. *Int. J. Legal. Med.* **2019**, *133*, 1581-1592.

31. Matuszewski, S.; Mądra-Bielewicz, A. Post-mortem interval estimation based on insect evidence in a quasi-indoor habitat. *Sci. Justice* **2019**, *59*, 109-115.

32. Mashaly, A.; Al Khalifa, M.; Al Qahtni, A.; Alshehri, A. Analysis of insects colonised on human corpses during autopsy in Riyadh, Saudi Arabia. *Entomol. Res.* **2020**, *50*, 351-360.

33. Pérez-Marcos, M.; Arnaldos-Sanabria, M.I.; García, M.D.; Presa, J.J. Pérez-Marcos, M.; Arnaldos-Sanabria, M.I.; García, M.D.; Presa, J.J. Examining the sarcosaprophagous fauna in a natural mountain environment (Sierra Espuña, Murcia, Spain). *Ann. Soc. Entomol. Fr.* **2016**, *52*, 264–280.

34. Moemenbellah-Fard, M.D.; Keshavarzi, D.; Fereidooni, M.; Soltani, A. First survey of forensically important insects from human corpses in Shiraz, Iran. *J. Forensic Leg. Med.* **2018**, *54*, 62-68.

35. Adair, T.W.; Kondratieff, B.C. The occurrence of *Nitidula flavomaculata* (Coleoptera: Nitidulidae) on a human corpse. *Entomol. News* **1996**, *107*, 233-236.

36. Bonacci, T.; Mendicino, F.; Carlomagno, F.; Bonelli, D.; Marchetti, M.G.; Vicenzi, A.; Scapoli, C.; Pezzi, M. Necrodes littoralis (Coleoptera: Silphidae) visiting and breeding on a carcass in Italy. *Trop. Biomed.* **2022**, *39*, 203-208.

37. Bonacci, T.; Mendicino, F.; Bonelli, D.; Carlomagno, F.; Curia, G.; Scapoli, C.; Pezzi, M. Investigations on arthropods associated with decay stages of buried animals in Italy. *Insects* **2021**, *12*, 311.

38. Oliva, A. Insects of forensic significance in Argentina. *Forensic Sci. Int.* **2001**, *120*, 145-154.

39. Jung, J.B.; Yoon, M.H. A study on the arthropod succession in exposed pig carrion. *J. Life Sci.* **2008**, *18*, 1400-1409.
